# Proteinuria as a predictor of complications of pre-eclampsia

**DOI:** 10.1186/1741-7015-7-11

**Published:** 2009-03-24

**Authors:** G Justus Hofmeyr, Michael Belfort

**Affiliations:** 1 Effective Care Research Unit, Eastern Cape Department of Health, Universities of the Witwatersrand and Fort Hare, South Africa; 2 Dept of Obstetrics and Gynecology, University of Utah School of Medicine, Salt Lake City, UT, USA; 3 Dept of Perinatal Research and Fetal Therapy, HCA Healthcare, Nashville TN, USA

## Abstract

Proteinuria is a defining criterion for the diagnosis of pre-eclampsia. The amount of protein lost per day has been thought by some to predict both maternal and fetal outcome. The systematic review of 16 primary papers including over 6700 patients by Thangaratinam and colleagues published this month in BMC Medicine suggests otherwise. This finding may influence our management of pre-eclampsia.

## Commentary

Proteinuria has been proposed and studied as both an indicator of severity of disease and as a predictor of outcome in pre-eclampsia. Many clinicians still make major management decisions based on the degree of proteinuria in such patients. The systematic review by Thangaratinam and colleagues [1] published this month in BMC Medicine suggests however that proteinuria is a poor predictor of either maternal or fetal complications in women with pre-eclampsia, and provides information that may have significant clinical implications.

Pre-eclampsia affects 2 to 3% of all pregnancies and is responsible for about 60,000 maternal deaths every year, mainly in poor countries [2]. Annually only 10 of these deaths occur in the UK [3], approximately 40 to 50 in the USA [4], while in comparison more than 200 occur in South Africa [5]. The only known cure for pre-eclampsia is delivery of the placenta. This creates a conflict of interest between the individuals on either side of the placenta: the mother stands to benefit from early delivery, while the baby may suffer complications of prematurity if born too early. Conservative management of pre-eclampsia to gain time for the baby to mature inevitably places the mother at risk [6]. Pre-eclampsia is usually a progressive disease, but the rate of progression and the occurrence of catastrophic complications such as eclampsia, cerebrovascular accident, severe HELLP syndrome, pulmonary edema or renal failure are difficult to predict. Any marker which could reliably predict the likelihood of serious complications would be very valuable for helping choose the optimal time for delivery.

Proteinuria is a defining dysfunction of pre-eclampsia [7]. Quantitation of a timed collection has been the gold standard for many decades and is expressed as the amount of protein excreted in the urine per unit time. Twenty-four-hour specimens have been traditionally used, but more recently 12-hour collections (and even 2-hour collections) have been validated [8]. The urinary protein:creatinine ratio is used in some institutions instead of a timed protein collection [9], with some finding it to be equally useful in determining pathologic proteinuria with the advantage of not requiring a timed collection, while others have not been as confident [10]. A 24-hour collection remains the standard of care in the USA [7].

The severity of the proteinuria in pre-eclampsia has been regarded by some as a predictor of adverse outcomes for the mother [11]. Others have been less sanguine about the relationship [12]. A reliable correlation between the level of proteinuria and severity of pre-eclamptic complications would be extremely valuable for clinical decision making.

The review by Thangaratinam et al [1] reported in this issue sets a new standard for systematically searching for, evaluating and aggregating the results of studies of this kind. The results are disappointing in that the correlation found between level of proteinuria and severity of clinical disease was insufficiently reliable to be clinically useful. The authors reported that from a fetal point of view, the only statistically significant findings were that proteinuria of 5 g/24 h in a timed specimen, or 1+ and 3+ in a dipstick specimen, predicted stillbirth with a likelihood ratio for the positive result of 1.3 to 2.3 ('little useful' to 'somewhat useful'). Maternal outcomes fared equally poorly. The same group of authors has previously reported on another biochemical marker, serum urate, with similarly disappointing results [13].

Despite the rigor and efforts to determine the quality of the studies included in the current review, practice differences, equipment changes, and definitions of pre-eclampsia could have influenced the diagnosis (and management) of pre-eclampsia over the time period of the studies used. Thirty years ago changes in systolic pressure and diastolic pressure during gestation were being used to define pre-eclampsia (the so-called 30/15 rule) and if the diagnosis of pre-eclampsia is differently defined in different studies the validity of the result may be diminished.

A very important potential confounding factor to consider in studies of the kind reviewed, is that the test result (in this case severe proteinuria), particularly in the earlier studies, may have dictated management. In the USA at least, proteinuria of 5 g or more per 24 hours is one of the diagnostic criteria for severe pre-eclampsia [7]. If women were delivered earlier as a result of a positive test for severe proteinuria then that test cannot be stated to have been used to predict outcome, since its result was used to intervene and thus influence the outcome. Earlier delivery precipitated by a positive test result may, for example, reduce maternal complications (leading to an underestimation of the predictive value of the test), or increase perinatal morbidity due to prematurity, leading to an overestimation. The test would technically have an association with the outcome, rather than a predictive capability.

Despite these limitations, this metanalysis appears to confirm what clinicians have suspected for a long time. The degree of proteinuria alone does not have a strong association with adverse outcome. Maternal and fetal clinical condition and gestational age, complemented by hematologic and biochemical parameters, should for the time being remain the primary determinants for timing delivery in women with pre-eclampsia.

As the results of observational studies may systematically over- or underestimate the predictive value of tests as discussed above, a randomized trial of knowledge versus no knowledge of the level of proteinuria to guide management would be justified.

## Competing interests

The authors declare that they have no competing interests.

## Authors' contributions

MAB and GJH drafted the manuscript and MAB revised it for important intellectual content. MAB and GJH have both given final approval of the version to be published. Each author has participated sufficiently in the work to take public responsibility for the content.

## Pre-publication history

The pre-publication history for this paper can be accessed here:


